# Quinolone‐induced radiation recall dermatitis in breast cancer patient

**DOI:** 10.1111/tbj.13756

**Published:** 2020-01-30

**Authors:** Stefana Rafiroiu, Andrew Vassil, Stephanie A. Valente

**Affiliations:** ^1^ Heritage College of Osteopathic Medicine Warrensville Heights Ohio; ^2^ Department of Radiation Oncology Cleveland Clinic Cleveland Ohio; ^3^ Department of General Surgery, Division of Breast Surgery Cleveland Clinic Cleveland Ohio

1

A 58‐year‐old woman presented with acute onset skin changes to her left breast. She has a history of left breast ductal carcinoma in situ, 7 mm in size, estrogen positive treated 5 years prior. She underwent a lumpectomy with clear margins, followed by adjuvant whole breast radiation (5000cGy ×25 fractions, 1000cGy ×5 fractions tumor bed boost) without complications. Expected levels of hyperpigmentation and erythema (CTCAE v5 Gr1) upon completing radiation were resolved by her 1‐month follow‐up. She was started on tamoxifen and then switched to exemestane due to elevated triglycerides. She tolerated this well and maintained on this regimen.

**Figure 1:**
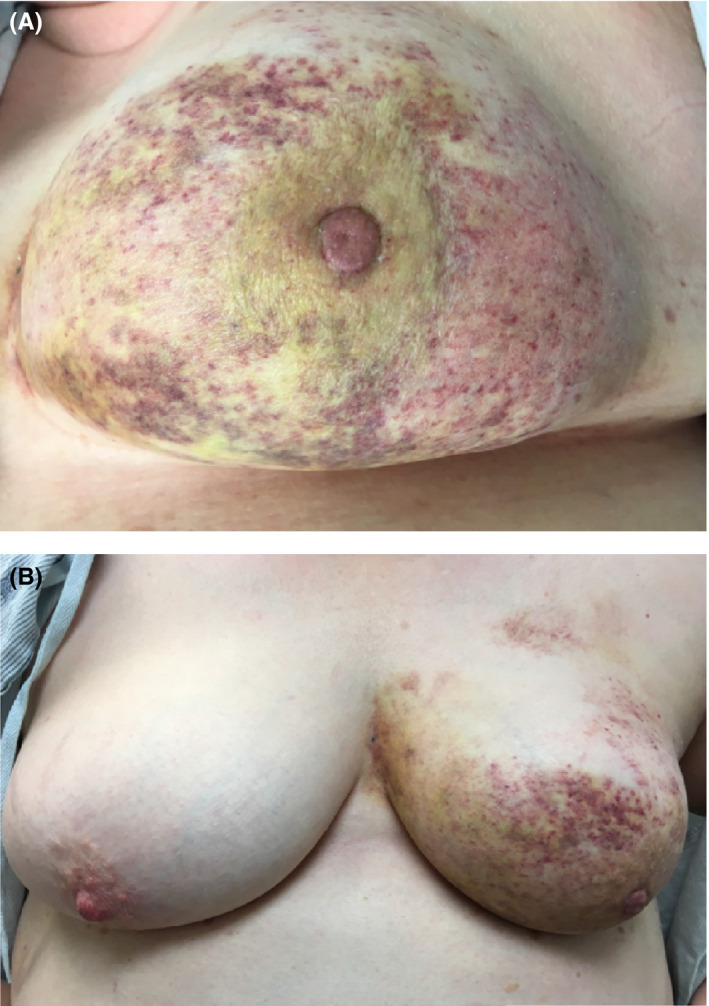
LEFT breast with acute, painless, diffuse brusing and rash

**Figure 2:**
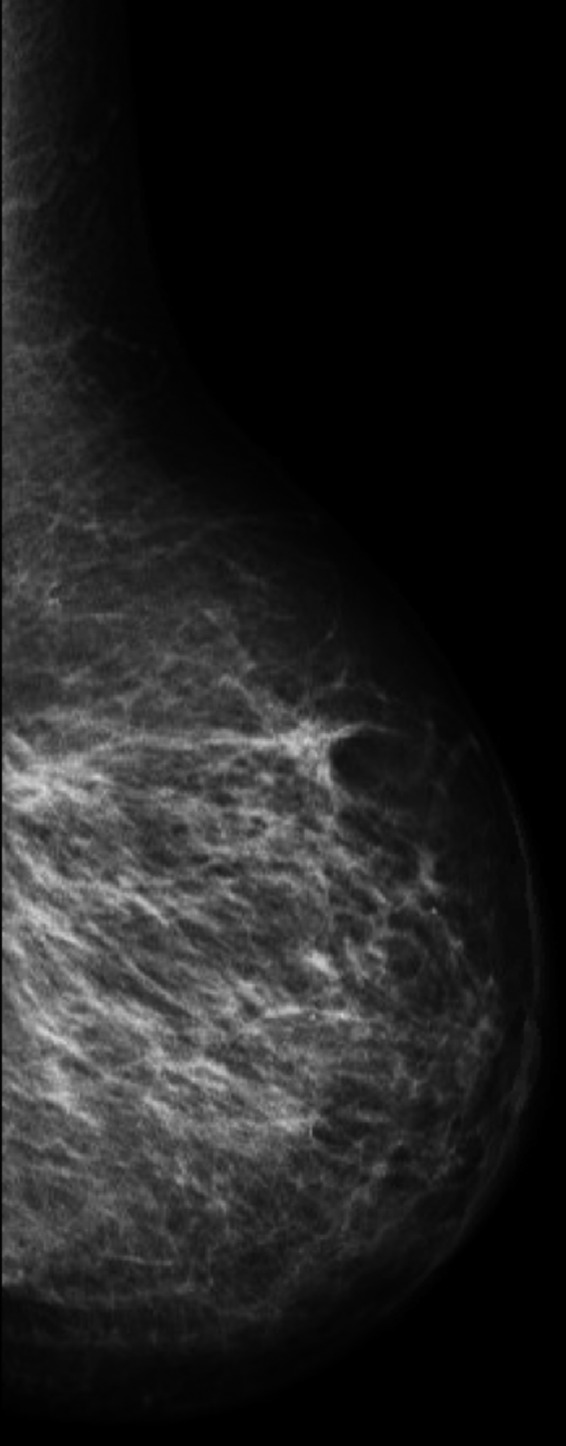
Diagnostic mammogram showed only diffuce, nonspecific skin thickening, no underling mass or asymmetry

She had been feeling well, no trauma, but developed sudden, severe painless bruising to her left breast. Examination revealed diffuse skin discoloration and bruising of her left breast with changes limited to, yet not entirely encompassing the radiation field, most significantly at the dependent portion of the breast (Figure 1). She was afebrile with no leukocytosis. Mammogram and ultrasound reported diffuse whole breast skin thickening, with no suspicious masses or calcifications (Figure 2). A skin punch biopsy showed fibrosis and vascular ectasia with thrombosis, and atrophic epidermis with mild patchy spongiosis on background of radiation changes.

History revealed an episode of bronchitis treated 2 months prior to her presentation treated with a course of ciprofloxacin followed by levofloxacin. Radiation recall dermatitis (RRD) was suspected, and within 2 weeks of starting clobetasol steroid cream, the skin changes resolved completely.

Radiation recall dermatitis is an acute inflammatory skin reaction within a previously irradiated area that is induced by administration of a drug. Chemotherapy accounts for the majority of RRD cases but additional eliciting drugs include statins and antibiotics such as  azithromycin, nitrofurantoin, and quinolones . RRD typically develops within weeks of the first dose but can occur years after, with an average of forty days from the end of radiation. RRD differs from radiosensitization, which is less severe and appears under seven days from radiotherapy. Our case developed RRD over 5 years after radiation and 2 months after quinolone use, the longest time among reported quinolone‐induced RRD.

Patient history helps identify specific fields of prior radiation and the eliciting drug. Physical examination typically includes edematous maculopapular eruption with desquamation localized to radiated skin, and rarely ulceration or necrosis. Biopsy excludes malignancy by yielding atrophic epidermis, vascular degeneration, papillary dermis edema, or mononuclear cell infiltration.

RDD treatment includes discontinuation of the eliciting drug and topical corticosteroids. Mild cases can resolve spontaneously with cessation of causative agent. In cases of cancers with a high rate of reoccurrence or in patients without alternative treatment, the eliciting drug may be continued. Rechallenging with the drug may elicit no reaction, milder, or more severe reactions.

Radiation recall dermatitis should be a differential diagnosis for erythematous edematous rashes in patients with a history of radiotherapy and antimicrobial therapy. Its diagnosis becomes more relevant as cancer patient survival increases.

